# Skin Microbiota, Circulating Metabolic Biomarker, Epilepsy: A Mendelian Randomization Analysis

**DOI:** 10.3390/cimb46120833

**Published:** 2024-12-11

**Authors:** Yongheng Wang, Tianyi Liu, Shangying Wu, Jiawei Sun, Mingdao Mu, Zhiyuan Yang

**Affiliations:** 1 School of Artificial Intelligence, Hangzhou Dianzi University, Hangzhou 310018, China; wyh520520520@163.com (Y.W.); jwsun@hdu.edu.cn (J.S.); 2 School of Biomedical Sciences, The Chinese University of Hong Kong, Hong Kong 999077, China; wu_shangying@163.com; 3 School of Medicine, Southeast University, Nanjing 210009, China

**Keywords:** epilepsy, Mendelian randomization analysis, skin microbiota, circulating metabolic biomarker

## Abstract

Epilepsy is a neurological disorder characterized by recurrent, unprovoked seizures. Currently, the associations among skin microbiota, circulating metabolites, and epilepsy are still not well studied. In this study, we applied univariate and two-step Mendelian randomization analysis using single nucleotide polymorphisms as instrumental variables to analyze the possible associations. Five skin microbiota (asv022, asv008, bacillales, clostridiale, and micrococcaceae) and four circulating metabolites were found to be associated with epilepsy. Our results also showed that leucine level (*p*-value = 0.0487, OR = 1.067) and glutamine level (*p*-value = 0.0372, OR = 1.065) show a slight increased association with epilepsy, while phospholipids in medium LDL (*p*-value = 0.0302, OR = 0.9308) and ratio of saturated fatty acids (*p*-value = 0.0309, OR = 0.9017) appear to show a slight inverse correlation with epilepsy. In addition, the heterogeneity test and horizontal pleiotropy analysis indicated these results are robust in different situations. By mapping analysis, 54 genes were associated with at least two metabolites. By functional analysis, these genes were enriched in the immune-related pathways, which may have systemic effects on brain function. Our results provide a novel insight for understanding possible mechanisms of some epilepsy associations, which by further study might provide avenues for therapy research.

## 1. Introduction

Neurological disorders are the leading cause of disability and the second leading cause of death worldwide. As one of the neurological diseases, epilepsy has recurrent and transient seizure characteristics, and affects approximately 0.67% of people worldwide [1]. Studies have shown that about 13 million people are disabled each year due to epilepsy, leading to a reduction in life expectancy [2]. According to Falco-Walter et al. [3], epilepsy can be divided into four types: focal, generalized, combined, and unknown. The etiology of epilepsy is also diverse, including structural, genetic, infectious, metabolic, and immune causes [4]. The seizure mechanism of epilepsy may involve several small distributed hyperexcitable network regions that undergo frequent electrophysiological changes, causing the brain to transition into various seizure states. When these electrophysiological changes slowly expand and involve more normal brain areas, they can lead to clinical seizures [5]. The mechanisms of action of antiepileptic drugs have distinct characteristics and can be divided into the following four types: (1) regulation of sodium, calcium, or potassium channels; (2) alterations in inhibition via actions on *GABA* receptors or on *GABA* synthesis, reuptake, or degradation; (3) decreased synaptic excitation via actions on ionotropic glutamate receptors; and (4) modulation of neurotransmitter release via presynaptic mechanisms, with action on glutamate release being most relevant [6]. Studies have shown that drug resistance mechanisms in epilepsy include the failure of drugs to reach their targets, alterations in drug targets, and the drugs missing their real targets [7].

Both genetic and environmental factors play significant roles in the development and manifestation of epilepsy. The influence can vary widely from person to person, depending on the underlying genetic predispositions, and exposure to environmental triggers [8]. However, epilepsy is a highly heterogeneous disorder, with significant differences in pathogenesis, clinical presentation, and treatment, making microbiome studies more complex and involving multiple variables [9]. Skin microbiota is one of these environmental factors and it refers to the community of microorganisms that reside on the skin surface. It includes many microorganisms, such as bacteria, fungi, viruses, and mites. These microorganisms form a complex ecosystem that plays an essential role in maintaining skin health, immune function, and protection against diseases [10]. However, little research has reported on how the skin microbiota status has an impact on the occurrence of epilepsy. Thus, we hypothesize that changes in the skin microbiota may have an effect on the development and onset of epilepsy. The relationship between metabolites and the human microbiota is closely interconnected. One study showed that the gut microbiome is associated with differences in triglyceride and high-density lipoprotein (HDL) levels, probably reflecting the person’s dietary intake [11], while the influence of the gut microbiome on low-density lipoprotein (LDL) cholesterol or total cholesterol levels is weaker compared to triglycerides and HDL [12]. Research has identified three distinct metabolite–microbe clusters: one dominated by Cutibacterium, linked to hydrophobic elements of the skin barrier; one associated with the Staphylococcus genus, relating to amino acids important for water retention and pH regulation of the skin surface; and one characterized by Streptococcus, independent of any specific metabolomic profile [13]. Therefore, we can infer that a change in the skin microbiota may lead to corresponding changes in circulating metabolites.

In addition, epilepsy is often associated with altered energy metabolism, particularly in the brain [14]. Seizures involve intense electrical activity in neurons, which can cause significant energy demands and metabolic stress. Changes in circulating metabolites related to energy production have been observed in people with epilepsy [15]. The upregulation or downregulation of individual circulating metabolites to some extent reflects the differences in genetic expression and changes in the body environment. Previous studies suggest that plasma metabolites may be used as biomarkers for diseases, providing a clinical application basis for the treatment of patients with temporal lobe epilepsy [16]. These metabolites may be related to the drug resistance process in patients with refractory temporal lobe epilepsy. This indicates that changes in circulating metabolites in blood may cause or inhibit the development of epilepsy.

To study the association among skin microbiota, circulating metabolites, and epilepsy, a Mendelian randomization (MR) analysis was conducted to explore whether changes in the skin microbiota can cause changes in blood circulation metabolites, leading to the occurrence of epilepsy. MR is a method in epidemiology that uses genetic variants, for example, single nucleotide polymorphisms (SNPs), as instrumental variables (IVs) to assess the causal relationship between an exposure (skin microbiota) and an outcome (epilepsy) [17]. Since genetic variants are randomly assigned at conception, MR mimics the randomization process in clinical trials, helping to reduce confounding and reverse causation issues common in observational studies. MR provides a way to infer causality by analyzing how genetic predispositions for certain exposures affect health outcomes, offering insights into disease mechanisms and potential therapeutic targets.

In MR analysis, a mediator is an intermediate variable that lies on the causal pathway between the exposure and the outcome [18]. It represents a mechanism through which the exposure exerts its effect on the outcome. Mediators are important because they help to understand the biological pathways of how a certain exposure influences the outcome [19]. In this study, we use circulating metabolites as mediators to investigate whether skin microbiota exposure has a causal effect on epilepsy, and use blood circulation metabolic markers as mediators to explore the percentage of mediation.

## 2. Materials and Methods

### 2.1. Data Sources

**Figure 1:**
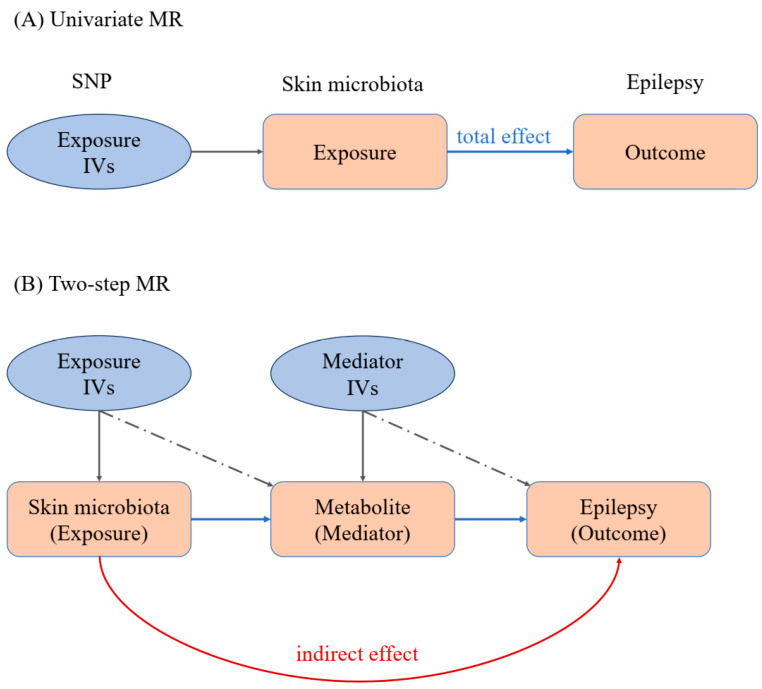
MR workflow of our work. (**A**) Univariate Mendelian randomization analysis; (**B**) two-step Mendelian randomization analysis. IVs: instrumental variables. The IVs are shown in light blue ellipse, while exposure and outcome are shown in pink block.

The genome-wide association analysis (GWAS) data for skin microbiota were obtained from the GWAS Catalog database [20]. The GWAS Catalog website directory provides a consistent, searchable, visual, and free SNP trait association database that can be easily integrated with other resources for scientists, clinicians, and other users worldwide to access. GWAS data of epilepsy is sourced from the FinnGen database [21]. The FinnGen database is a large-scale research project focused on personalized medicine and collected genomic data from about 500,000 Finnish biobank donors. GWAS data could provide a series of important indexes, such as SNP, effect size (*β* value), standard error, effect allele, non-effect allele, and allele frequency. The circulating metabolic biomarkers of blood in this study were obtained from Karjalainen et al. [22]. The overall experimental process is shown in Figure 1.

### 2.2. Univariate MR Analysis

Mendelian randomization is a method used in epidemiological research that utilizes genetic variation as an instrumental variable to evaluate the causal relationship between exposure and outcomes. This study mainly examines the inverse variance weighted (IVW) method [23]. The concept of instrumental variables (IVs) originated from econometrics and is a means of inferring causal relationships in the absence of confounding factors. In this study, SNP was selected as the instrumental variable from GWAS data. To ensure compliance with the IV hypothesis, the following criteria were applied in the SNP selection process:

(1) Using a strict genome-wide significance threshold (*p* ≤ 5 × 10^−5^), SNPs were extracted from GWAS exposure data, establishing a strong association with exposure [24].
(2) Remove SNPs with linkage disequilibrium (LD). When the distributions of two SNPs are correlated rather than independent, LD occurs, which violates the assumption of random classification and may bias the results. In this study, we used the “TwoSampleMR (version: 0.5.7)” package to screen SNPs with parameters *r*^2^ = 0.001 and *kb* = 10,000, which means that SNPs with *r*^2^ = 0.001 are removed within the 10,000 kb region surrounding the most significant SNP to eliminate potential LDs.
(3) Screen out SNPs related to obsessive–compulsive disorder from the relevant GWAS data of the results. After initial SNP screening, calculate the total F-value of IVs to assess the strength of instrumental variables and mitigate potential bias caused by weak IVs. If the F value exceeds 10, it can be considered a strong instrumental variable.

### 2.3. Two-Step MR Analysis

Two-step Mendelian randomization is the application of Mendelian randomization with three or more samples, mainly used to evaluate whether the mediator has a mediating effect on the overall effect. The first step is to use instrumental variables to evaluate whether exposure has a causal effect on the mediator; the second step is to use the instrumental variables of the mediator (excluding the first step) to evaluate if the mediator has a causal effect on the outcome. The effect value of exposure on the outcome is the total effect value, the effect value of mediation on the outcome is the direct effect value, and the indirect effect value is the difference between the two factors.

### 2.4. Analysis of Heterogeneity

We conducted sensitivity analysis on the obtained data to evaluate the reliability and validity of the results. This process includes heterogeneity and horizontal pleiotropy analysis. We used the IVW method, MR Egger regression, and weighted median estimation as sensitivity analyses to evaluate the robustness of the MR results from the “TwoSampleMR (version: 0.5.7)” software package. MR Egger regression is a weighted linear regression that examines the correlation between SNP outcomes and SNP exposure. Weighted median estimation is the median of MR scores, weighted proportionally to its accuracy. Conduct sensitivity analysis and statistical testing to evaluate the validity of hypotheses. Cochran’s Q test was used to estimate SNP heterogeneity, with a *p*-value ≥ 0.05 indicating no heterogeneity. When there is significant heterogeneity between SNPs, a random effects model is used; otherwise, a fixed effects model will be used.

### 2.5. Analysis of Horizontal Pleiotropy

We used the “Mendelian Randomization Pleiotropy RESidual Sum and Outlier” (MR-PRESSO) method [25] to detect potential outliers, and calculated causal estimates after removing identified outliers. MR-PRESSO global testing and MR Egger intercept are used to screen for outliers and evaluate potential level pleiotropy. The MR Egger intercept represents horizontal pleiotropy (intercept *p*-value ≤ 0.05), and the slope can robustly estimate the causal effects when pleiotropy exists. When a significant level of pleiotropy is detected, the MR-PRESSO outlier test is used to correct for pleiotropy by removing or reducing the influence outlier (*p*-value ≤ 0.05 in MR-PRESSO global test). In addition, the MR-PRESSO distortion test was used to identify significant distortions in causal estimates before and after removing outliers. Use the “MR-PRESSO (version: 1.0)” package for MR-PRESSO global, outlier, and distortion testing.

### 2.6. Analysis of Effect Value

**Figure 2:**
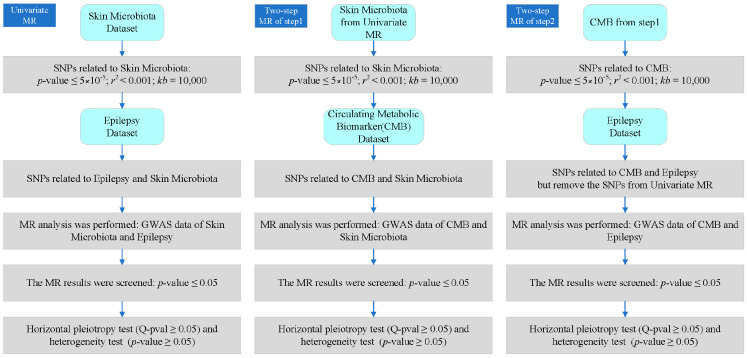
Detailed process and criteria of our analysis. CMB: circulating metabolic biomarker.

After the heterogeneity and horizontal pleiotropy tests, the effect value analysis was conducted. The total effect was the outcome effect value of direct exposure, the indirect effect value was the effect value of exposure that acted on the outcome only through mediation, and the direct effect value was the effect value of exposure that acted on the outcome without mediation (this is the difference between the total effect and the indirect effect). In addition, indirect and total effect values were used to calculate the mediation-mediated percentage. The following calculation formula was based on a previous study of deciphering effect values of MR [26]. The details of each step are shown in Figure 2.

indirect effect = step 1 effect × step 2 effect

direct effect = total effect − indirect effect

mediator percent = indirect effect/total effect

### 2.7. Associated Genes Analysis

In this study, we used SNPs as the instrumental variable (exposure) to calculate the association between skin microbiota, circulating metabolites, and epilepsy. In order to reveal the possible underlying mechanism, SNP-gene mapping analysis was conducted by the SNP2GENE tool (version: v1.6.1) in the FUMA website “https://fuma.ctglab.nl (accessed on 6 October 2024)” [27]. In this tool, the genes were obtained through three methods, including genomic position mapping, expression quantitative trait loci (eQTL) mapping, and Chromatin Interaction mapping. The common genes among four circulating metabolic biomarkers were identified. To analyse the functions of the common genes, we applied the tool Sangerbox (version: 3.0) [28], a user-friendly online platform, to identify significant enriched metabolic pathways. The functions of genes in these pathways were further discussed to identify their possible mechanism in epilepsy.

## 3. Results

### 3.1. Data Classfication

**Figure 3:**
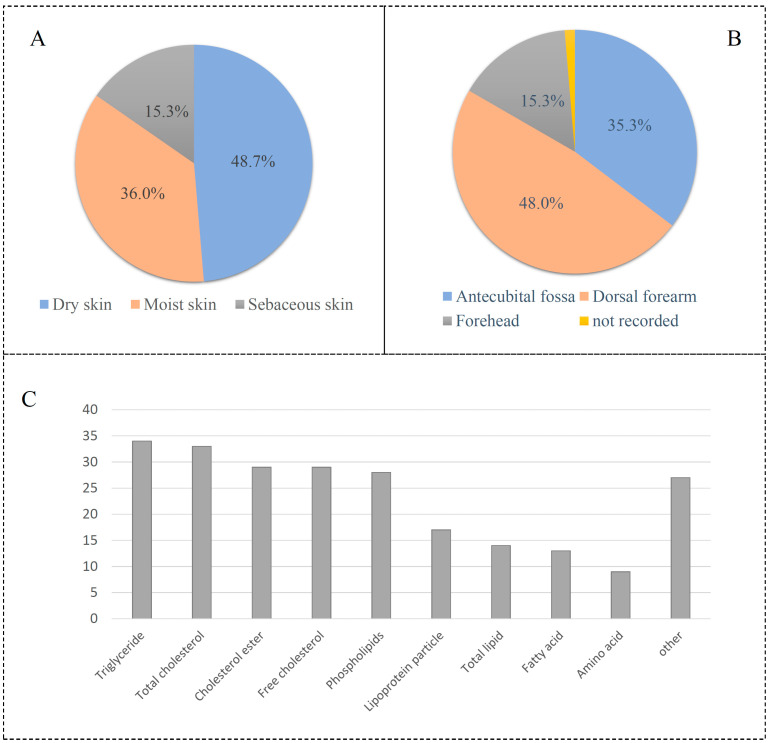
Description of skin microbiota and circulating metabolic biomarkers. (**A**) Skin type of microbiota; (**B**) skin location of microbiota; (**C**) distribution of circulating metabolic biomarkers.

The GWAS data for epilepsy consist of 12,891 affected groups and 312,803 control groups. The data for skin microbiota consist of 150 univariate microbial characteristics for dry, moist, and sebaceous skin, with a sample size of 597 individuals from Europe (Table S1). Results showed that 15.3% of skin types are dry, 36.0% are moist, and 48.7% are sebaceous (Figure 3A). A set of 48.0% of skin microbiotas is from the dorsal forearm, while 35.3% of skin is from the antecubital fossa (Figure 3B). The data for circulating metabolic markers consist of 233 blood metabolites, with samples from Asian and European populations (Table S2). These metabolite markers included 34 triglyceride indexes, 29 cholesterol esters, 28 phospholipids, and so on (Figure 3C).

### 3.2. Univariate MR Results

To evaluate the impact of 150 skin microbiota on epilepsy, two types of MR analysis, including univariable MR and two-step MR, were performed for these skin microbiota and epilepsy. Three indexes, including *p*-value, odds ratio (OR), and confidence interval (CI), were calculated by the inverse variance weighted (IVW) method to evaluate their association. Results indicated that nine microbiota exhibited causal effects on epilepsy with the cutoff statistical *p*-value ≤ 0.05 in the IVW method. These microbiotas included asv008, asv022, asv039, asv042, bacillales, clostridiales, micrococcaceae, neisseriaceae, and staphylococcus (Table 1).

The MR analysis of neisseriaceae showed results of *p* = 0.0291, OR = 1.0106, and 95% CI = (1.001–1.025), suggesting that a low level of neisseriaceae was associated with a lower probability of epilepsy. For staphylococcus, MR analysis yielded the results *p* = 0.0287, OR = 1.0106, and 95% CI = (1.001–1.020), indicating that a high level of staphylococcus increased the probability of epilepsy. The results for clostridiales were *p* = 0.0428, OR = 1.0102, and 95% CI = (1.000–1.020), indicating that clostridiales were associated with an increased probability of epilepsy.

### 3.3. Two-Step MR Results

Subsequently, we conducted a two-step MR analysis to investigate how skin microbiota affect epilepsy through the metabolites. In the first step, we applied MR analysis between skin microbiota and circulating metabolic biomarkers. Results showed that five microbiotas (asv008, asv022, bacillales, clostridiales, micrococcaceae) were found to be associated with four circulating metabolites (GCST90301965, GCST90301994, GCST90302078, GCST90302151) (Table 2). GCST90302078 showed a connection with two microbiotas (asv008 and clostridiales); thus, only four microbiotas remained in step 2. Three microbiota are from the dry skin of the dorsal forearm, while the other two were from moist skin and sebaceous skin, respectively. The MR analysis of asv022 showed results of *p* = 0.0011, OR = 0.996, and 95% CI = (0.9936–0.9984), suggesting that asv022 has a slight protective factor for epilepsy.

In step 2, we applied MR analysis to circulating metabolic biomarkers and epilepsy. The above-obtained four metabolites (glutamine level, leucine level, ratio of saturated fatty acid, phospholipids in medium LDL) were found to be associated with epilepsy. The results for GCST90302078 showed *p* = 0.0309, OR = 0.9017, and 95% CI = (0.8209–0.9905), indicating that “Ratio of saturated fatty acid” was associated with a decreased probability of epilepsy. The odd ratio (OR) value in the first step ranges from 0.9939 to 0.9998, while the OR value in the second step ranges from 0.8209 to 0.9905. This result indicated that the metabolites have a higher impact on epilepsy than any minimal impact of skin microbiota.

### 3.4. Results of Heterogeneity Analysis

To assess whether the genetic SNPs (instrumental variables) show a consistent pattern of effect when influencing the outcome variable, heterogeneity testing analysis was conducted in this study. By the IVW and MR Egger methods, the Q value, degree of freedom, and *p*-value of the Q test (Q-pval) were calculated. If the *p*-value of the heterogeneity test is less than the predetermined significance level (*p*-value ≤ 0.05), statistical heterogeneity is considered to exist. After removing factors with heterogeneity test results less than 0.05, the summary of the heterogeneity test results is shown in Table 3. The exposures with the top three Q_pval were clostridiales, micrococcaceae, and bacillales. For clostridiales, the Q_pvals with the two methods are 0.200 and 0.203, respectively. For micrococcaceae, the Q_pval with the MR Egger method is 0.214, while the Q_pval with the IVW method is 0.243. For bacillales, the Q_pval with the MR Egger method is 0.139, while the Q_pval with the IVW method is 0.162. Overall, the heterogeneity test results for these five skin microbiota were all greater than 0.05, indicating no significant heterogeneity in the data for these factors. This result indicated that our MR analysis results are not influenced by other factors, such as confounding factors, study design differences, and measurement errors.

### 3.5. Horizontal Pleiotropy

In MR analysis, we also need to evaluate if genetic SNPs (instrumental variables) may directly affect outcome variables in addition to being affected by exposure. This direct effect is called horizontal pleiotropy, which may affect the assumption of MR analysis that instrumental variables are only correlated with outcome variables through exposures. The MR Egger method was applied to the horizontal pleiotropy test, where the intercept value and its associated *p*-value were used to assess the presence of horizontal pleiotropy. The summary of the horizontal pleiotropy test results is presented in Table 4. The circulating metabolic biomarkers with the top two *p*-values are GCST90302078 and GCST90301965. For GCST90302078, the intercept was 0.0003 and *p*-value was 0.8161. For GCST90301965, the intercept was −0.0005 and *p*-value was 0.8182. Overall, the intercepts of these four metabolites were small in magnitude, and the *p*-values were greater than 0.05, indicating that no significant evidence of horizontal pleiotropy was present in these factors.

### 3.6. Effect Value

After the heterogeneity and horizontal pleiotropy analysis, we also conducted the effect value analysis. The results are shown in Table 5. The total effect of asv022 against epilepsy is −1.149%, including an indirect effect of −0.026% through GCST90301994 and a direct effect of −1.123%. The total effect of bacillales against epilepsy is 1.183%, including an indirect effect of 1.158% through GCST90302151 and a direct effect of 0.025%. All of these effect values are still close to zero, indicating that any influence of skin microbiota on epilepsy is minimal. The percentages mediated by three circulating metabolic biomarkers (GCST90301994, GCST90302151, GCST90301965) are around 2%. However, one metabolite GCST90302078 showed a very low mediation percentage of 0.08%. Two skin microbiotas have opposite effects on metabolites (GCST90302078). These results further suggest metabolites have some impact on the influence of epilepsy.

### 3.7. Associated Genes

**Figure 4:**
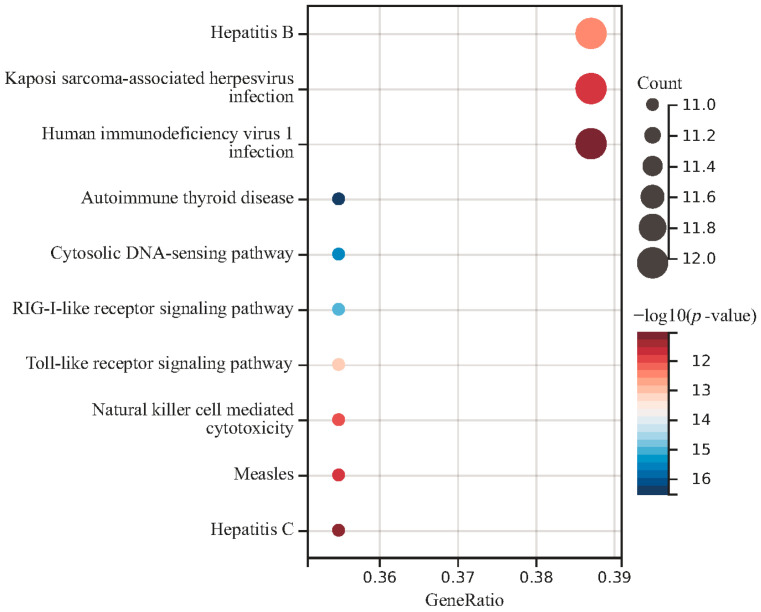
Function enrichment analysis of common genes in four circulating metabolic biomarkers.

To identify the associated genes with circulating metabolic biomarkers, we applied the FUMA website to identify the SNP-associated genes according to their genomic location. The genes associated with at least two metabolites are shown in Table S3, while the genes with at least three metabolites are shown in Table 6. Overall, we have identified 54 genes and 42 SNPs in the mapping analysis. The gene *MLXIPL* was found to be associated with four metabolites and the other 19 genes were associated with three metabolites. Gene *GCKR* is located nearby the SNP rs1260326 and this SNP was found to be associated with three metabolites by GWAS analysis. Moreover, Gene *APOE* was located near the SNP rs7412 and this SNP was found to be associated with metabolite GCST90302151. Function analysis of these 54 genes indicated that they are enriched in the immune-related pathways, such as autoimmune thyroid disease and the Toll-like receptor signaling pathway (Figure 4). The skin microbiota plays a key role in regulating the skin’s immune responses. Since the immune system and the central nervous system are closely interconnected, disruptions in immune responses triggered by skin microbiota imbalances might have systemic effects, potentially influencing brain function. These results suggest that epilepsy may be associated with the immune system.

## 4. Discussion

Epilepsy is characterized by several key features, primarily related to abnormal electrical activity in the brain, which leads to recurrent seizures [29]. Some individuals with epilepsy may experience cognitive difficulties or mood changes. Epilepsy is often considered to be a chronic disorder. While it can sometimes be controlled with medication or surgery, many individuals live with the condition for many years. The treatment of epilepsy focuses on controlling seizures, improving quality of life, and minimizing side effects. While epilepsy cannot always be cured, seizures can often be managed effectively with the right treatment plan.

The skin microbiota refers to the diverse community of microorganisms, including bacteria, fungi, viruses, and mites, that live on the skin [30]. The connection between the gut microbiota and various neurological disorders, including epilepsy, has been an area of growing interest and research. The skin microbiota and its relationship to epilepsy is less well understood. In this study, we have retrieved 150 skin microbiotas from the GWAS catalog database. By univariate MR analysis, nine microbiota (asv008, asv022, asv039, asv042, bacillales, clostridiales, micrococcaceae, neisseriaceae, staphylococcus) exhibited causal effects on epilepsy with the cutoff statistical *p*-value ≤ 0.05 in the IVW method. These bacteria, such as neisseriaceae, are common microbial pathogens of the central nervous system (CNS). In addition, bacteria in the staphylococcus genus may have an impact on the CNS. *Staphylococcus aureus* can directly infect the CNS following a neurosurgical procedure or effects through infection elsewhere in the body producing various toxins to affect the CNS [31]. If these inflammatory signals affect the brain, they could contribute to seizure activity or exacerbate existing epilepsy. These bacteria could potentially influence brain function or play a role in neurological disorders.

Metabolic disorders can influence neuronal excitability and increase the risk of seizures. The circulating metabolites are small molecules that are found in the bloodstream and are produced or transformed during metabolic processes within the body. These metabolites play key roles in various biological functions, serving as intermediates or end products of metabolism [32]. They come from a variety of sources, including the breakdown of nutrients from food, cellular activity, and interactions with the gut microbiota. The circulating metabolites are increasingly being studied for their potential associations with epilepsy, particularly in understanding its underlying mechanisms, identifying biomarkers, and developing new treatment strategies. Researchers are investigating how metabolic disturbances and alterations in certain metabolites in the bloodstream might influence the onset, frequency, and severity of seizures, as well as their potential to serve as biomarkers for epilepsy.

Mendelian randomization can be divided into univariate and two-step approaches [33]. Univariate MR is the standard and most commonly used form of Mendelian randomization. In this approach, the causal effect of a single exposure on an outcome is estimated using genetic variants as instrumental variables. Two-step MR is a specialized method used when a mediator lies between the exposure and the outcome. It is applied to assess whether the exposure affects the outcome through the mediator. In this study, we retrieved 233 circulating metabolic biomarkers from Karjalainen et al. and used these metabolites as mediators. By two-step MR analysis, four metabolic biomarkers (glutamine level, leucine level, ratio of saturated fatty acid, phospholipids in medium LDL) were found to be associated with epilepsy.

The heterogeneity and horizontal pleiotropy testing for these four metabolic biomarkers suggested that our MR analysis results are not influenced by study design differences or measurement errors. Glutamine levels are associated with epilepsy, primarily due to their role in the glutamine–glutamate–GABA cycle, which is critical for maintaining the balance between excitatory and inhibitory neurotransmission [34]. Disruptions in this balance, through altered glutamine metabolism, can contribute to seizure development and severity. Leucine, as a branched-chain amino acid, is involved in brain energy metabolism [35]. Disruptions in energy metabolism are known to play a role in seizure generation and epilepsy. Saturated fatty acids are a major component of the ketogenic diet, which helps reduce seizure activity by improving brain metabolism and stabilizing neuronal function [36]. Phospholipids are essential for maintaining neuronal membrane stability, regulating neurotransmitter release and cell signaling pathways that control brain excitability [37]. Based on the above description, these four molecules play a critical role in the pathology of epilepsy and may represent potential targets for therapeutic intervention. By SNP-gene mapping analysis, 54 genes were found to be associated with at least two circulating metabolic biomarkers. Some of these genes have been reported in epilepsy studies, for example, APOE. APOE is well known for its role in lipid transport, cholesterol metabolism, and maintaining neuronal integrity, particularly in the context of neurodegenerative diseases [38,39]. APOE has also been associated with increased neuroinflammation, which is a key factor in the development of epilepsy. The gene variants of APOE could contribute to epilepsy risk through this neuroinflammation mechanism, especially in the elderly population. As with many genetic factors implicated in this disease, the influences of these genes are likely part of a broader network of genetic interactions contributing to epilepsy.

Although we performed heterogeneity tests to exclude microbial communities or metabolic markers with significant heterogeneity, it is undeniable that our data were derived from European databases, with the majority of samples originating from Europe. However, there are certainly ethnic differences among countries within Europe. While the database predominantly consists of White individuals, the applicability of our model to other ethnic groups remains to be verified. In addition, we considered the singularity of the model linking skin microbiota, metabolic markers, and epilepsy. There may be other confounding factors that we have not accounted for, such as BMI, gender, and age, which could be significant influencing factors. However, our study did not include subgroup analyses based on these variables. Research has shown that high BMI is a risk factor for epilepsy in adolescents [40]. Individuals with high BMI tend to have larger body sizes or even obesity. Due to the effects of skin folds and sebum, changes in the skin microbiota are more pronounced in obese individuals compared to those with leaner body types. Therefore, developing more complex models to address these factors is an area we need to consider in future studies.

As the results show, because the skin is exposed to the air, the skin microbiota is influenced by many factors, which means its role in the model is relatively small (as indicated by the OR value, or effect size). Nevertheless, we should still take its role into account. Dividing the samples into multiple subgroups (e.g., obese vs. non-obese, male vs. female) for further discussion might yield more precise insights. Additionally, the etiology of epilepsy is extremely complex, and our study explores only one novel perspective on the potential causes or triggers of epilepsy. We must acknowledge that information on taking antiepileptic drugs was not provided in the metabolite samples. These factors may have a partial impact on our results. Fortunately, the epilepsy samples and metabolic marker samples are two separate datasets. The metabolic marker samples are relatively large, with over 100,000 European samples for each metabolite. This significantly reduces the potential influence of antiepileptic drugs on the experimental results.

In the context of skin microbiota, circulating metabolites, and epilepsy, the starting point is skin flora, the intermediary is metabolic markers, and the final outcome is epilepsy. We have established three associations through the five types of skin flora found and their corresponding relationships with four metabolite markers and epilepsy: (1) skin microbiota directly connects to epilepsy; (2) skin microbiota influences epilepsy by mediating metabolic markers; and (3) metabolic markers are directly linked to epilepsy. It is worth considering that changes in the skin microbiota may directly affect the development or onset of epilepsy, or may influence these processes through the mediation of metabolic markers. Additionally, we should directly consider that changes in metabolic markers may be associated with the onset or progression of epilepsy. Therefore, the clinical significance of this hypothesis is as follows: first, we can assess the possibility of epilepsy or disease occurrence by detecting changes in the four metabolic markers, in conjunction with the patient’s medical history; second, since detecting skin microbiota is simpler than detecting metabolic markers, changes in skin flora in patients could suggest the possibility of epilepsy; and third, simultaneous detection of both metabolic markers and skin microbiota could improve the accuracy of identifying epilepsy or other diseases.

We believe that these three approaches could also be applied to the prevention of epilepsy, even though we acknowledge that this remains a challenging task. For pathway analysis, whether as mediators or related to gene expression in vivo, we have established a pathway linking metabolic markers, SNPs, and genes. This extends the Mendelian randomization analysis, suggesting that some viruses, inflammatory conditions, or immune diseases may influence these metabolites, which could in turn affect epilepsy. While we acknowledge that this idea is far from being ready for clinical application, it does open the possibility for the treatment and prevention of epilepsy.

## 5. Conclusions

We have applied univariate and two-step Mendelian randomization analysis to investigate the associations among skin microbiotas, circulating metabolic biomarkers and epilepsy. We identified five microbiotas (asv008, asv022, bacillales, clostridiales, and micrococcaceae) as significant factors for epilepsy, while four metabolites (leucine level, glutamine level, phospholipids in medium LDL, ratio of saturated fatty acids) were considered as important mediators regulating epilepsy. By subsequent bioinformatics analysis, 54 genes were identified overlapping in four metabolites. Our results provide a novel insight for understanding possible mechanisms of some epilepsy associations, which by further study might provide avenues for therapy research.

## Figures and Tables

**Table 1:** Result of univariate MR analysis. OR: odds ratio; CI: confidence interval.

Skin Microbiota	Skin Type	Skin Location	Outcome	*p*-Value	OR	95% CI
asv008	sebaceous skin	forehead	epilepsy	0.0114	0.9899	0.982–0.998
asv022	moist skin	antecubital fossa	epilepsy	0.0083	0.9886	0.980–0.997
asv039	moist skin	antecubital fossa	epilepsy	0.0081	1.0101	1.002–1.018
asv042	moist skin	antecubital fossa	epilepsy	0.042	0.9924	0.985–0.999
bacillales	dry skin	dorsal forearm	epilepsy	0.0195	1.0119	1.002–1.022
clostridiales	dry skin	dorsal forearm	epilepsy	0.0428	1.0102	1.000–1.020
micrococcaceae	dry skin	dorsal forearm	epilepsy	0.0397	1.0111	1.000–1.021
neisseriaceae	sebaceous skin	forehead	epilepsy	0.0291	1.0133	1.001–1.025
staphylococcus	dry skin	dorsal forearm	epilepsy	0.0287	1.0106	1.001–1.020

**Table 2:** Result of two-step MR analysis. OR: odds ratio; CI: confidence interval.

Step	Exposure	Detail	Outcome	*p*-Value	OR	95% CI
Step 1	asv008	sebaceous skin in forehead	GCST90302078	0.0294	1.0003	1.0000–1.0051
	asv022	moist skin in antecubital fossa	GCST90301994	0.0011	0.9960	0.9936–0.9984
	bacillales	dry skin in dorsal forearm	GCST90302151	0.0316	0.9932	0.9932–0.9996
	clostridiales	dry skin in dorsal forearm	GCST90302078	0.0381	0.9969	0.9939–0.9998
	micrococcaceae	dry skin in dorsal forearm	GCST90301965	0.0291	0.9963	0.9930–0.9996
Step 2	GCST90301965	Glutamine level	epilepsy	0.0372	1.0655	1.0038–1.1311
	GCST90301994	Leucine level	epilepsy	0.0487	1.0675	1.0004–1.1391
	GCST90302078	Ratio of saturated fatty acid	epilepsy	0.0309	0.9017	0.8209–0.9905
	GCST90302151	Phospholipids in medium LDL	epilepsy	0.0302	0.9308	0.8723–0.9932

**Table 3:** Result of heterogeneity analysis. Q-pval: *p*-value of Q test.

Step	Exposure	Outcome	Method	Q Value	Q-df	Q-pval
Step 1	asv008	GCST90302078	IVW	38.9	49	0.850
	asv022	GCST90301994	IVW	36.1	41	0.690
	bacillales	GCST90302151	IVW	56.5	47	0.162
	clostridiales	GCST90302078	IVW	60.3	52	0.200
	micrococcaceae	GCST90301965	IVW	57.6	51	0.243
	asv008	GCST90302078	MR Egger	37.1	48	0.874
	asv022	GCST90301994	MR Egger	35.5	40	0.674
	bacillales	GCST90302151	MR Egger	56.5	46	0.139
	clostridiales	GCST90302078	MR Egger	59.1	51	0.203
	micrococcaceae	GCST90301965	MR Egger	57.6	50	0.214
Step 2	GCST90301994	epilepsy	IVW	204.6	189	0.207
	GCST90302151	epilepsy	IVW	226.2	207	0.171
	GCST90302078	epilepsy	IVW	149.0	151	0.530
	GCST90302078	epilepsy	IVW	149.0	151	0.530
	GCST90301965	epilepsy	IVW	246.9	224	0.141
	GCST90301994	epilepsy	MR Egger	204.1	188	0.200
	GCST90302151	epilepsy	MR Egger	226.1	206	0.160
	GCST90302078	epilepsy	MR Egger	149.0	150	0.507
	GCST90302078	epilepsy	MR Egger	149.0	150	0.507
	GCST90301965	epilepsy	MR Egger	246.8	223	0.131

**Table 4:** Result of horizontal pleiotropy analysis. SE: standard error.

Step	Exposure	Outcome	Intercept	SE	*p*-Value
Step 1	asv022	GCST90301994	0.0026	0.0035	0.4511
	bacillales	GCST90302151	0.0001	0.0037	0.9716
	asv008	GCST90302078	−0.0041	0.0031	0.1856
	clostridiales	GCST90302078	−0.0036	0.0035	0.3179
	micrococcaceae	GCST90301965	0.0001	0.0034	0.9798
Step 2	GCST90301994	epilepsy	0.0015	0.0021	0.4842
	GCST90302151	epilepsy	−0.0006	0.0023	0.7865
	GCST90302078	epilepsy	0.0003	0.0032	0.9161
	GCST90301965	epilepsy	−0.0005	0.0020	0.8182

**Table 5:** Result of effect value analysis. This table includes direct and indirect effect values.

Exposure	Mediator	Outcome	Total Effect	Indirect Effect	Direct Effect	Mediator Percentage
asv022	GCST90301994	epilepsy	−1.149%	−0.026%	−1.123%	2.26%
bacillales	GCST90302151	epilepsy	1.183%	0.025%	1.158%	2.15%
asv008	GCST90302078	epilepsy	−1.013%	−0.001%	−1.012%	0.08%
clostridiales	GCST90302078	epilepsy	1.015%	−0.001%	1.016%	−0.08%
micrococcaceae	GCST90301965	epilepsy	1.106%	−0.023%	1.129%	−2.10%

**Table 6:** List of 20 genes associated with at least three circulating metabolic biomarkers. The “NA” in this table indicated that the data is missing in this analsyis.

Gene	GCST90301965	GCST90301994	GCST90302078	GCST90302151	Number
*MLXIPL*	rs13246993	rs80189144	rs6460047	rs17145738	4
*AC109829.1*	rs1260326	rs1260326	NA	rs1260326	3
*EIF2B4*	rs1260326	rs1260326	NA	rs1260326	3
*GCKR*	rs1260326	rs1260326	NA	rs1260326	3
*PPM1G*	rs1260326	rs1260326	NA	rs1260326	3
*SNX17*	rs1260326	rs1260326	NA	rs1260326	3
*ZNF513*	rs1260326	rs1260326	NA	rs1260326	3
*APOC1*	NA	rs390082	rs429358	rs7412	3
*APOE*	NA	rs390082	rs429358	rs7412	3
*TOMM40*	NA	rs390082	rs429358	rs7412	3
*ARNTL2*	rs61915900	rs71534286	NA	rs71534286	3
*PPFIBP1*	rs74969426	rs74969426	NA	rs74969426	3
*PTHLH*	rs71534286	rs71534286	NA	rs71534286	3
*BAZ1B*	rs13246993	rs80189144	NA	rs17145738	3
*BCL7B*	rs13246993	rs80189144	NA	rs17145738	3
*FZD9*	rs13246993	rs80189144	NA	rs17145738	3
*TBL2*	rs13246993	rs80189144	NA	rs17145738	3
*BUD13*	NA	rs964184	rs964184	rs144018203	3
*ZNF259*	NA	rs964184	rs964184	rs964184	3
*APOA5*	NA	rs964184	rs964184	rs144018203	3

## Data Availability

All data can be found in the manuscript and Supplementary Materials.

## Supplementary Materials

The following supporting information can be downloaded at: https://www.mdpi.com/article/10.3390/cimb46120833/s1, Table S1: List of 150 skin microbiota in this study; Table S2: List of 233 circulating metabolic biomarkers in this study; Table S3: List of 54 genes associated with at least two circulating metabolic biomarkers.
